# Copolymerized carbon nitride nanoparticles for near-infrared II photoacoustic-guided synergistic photothermal/radiotherapy

**DOI:** 10.3389/fchem.2023.1124559

**Published:** 2023-01-13

**Authors:** Min Wu, Yuxin Huang, Xiaoyu Huang, Fu Wang, Xunbin Wei

**Affiliations:** ^1^ Department of Plastic and Reconstructive Surgery, School of Medicine, Shanghai Ninth People’s Hospital, Shanghai Jiao Tong University, Shanghai, China; ^2^ School of Biomedical Engineering, Shanghai Jiao Tong University, Shanghai, China; ^3^ Biomedical Engineering Department, Peking University, Beijing, China

**Keywords:** photothermal therapy, radiotherapy, copolymerized carbon nitride nanoparticles, photoacoustic, near-infrared II

## Abstract

Nanotheranostic agents that integrate diagnosis and treatment are promising for precision medicine, but they encounter some obstacles such as penetration depth and efficiency. In this study, novel carbon nitride-rose bengal nanoparticles (CN-RB NPs) with a graphite carbon nitride skeleton were synthesized by one-step thermal copolymerization. The enhanced absorption in the near-infrared-II region (NIR-II) endows CN-RB NPs with an excellent photothermal effect under 1064 nm laser irradiation, as well as an obvious photoacoustic signal for imaging *in vivo*. Interestingly, due to the introduced iodine element, CN-RB NPs exhibit enhanced radiation therapy, indicating that CN-RB NPs can achieve ideal therapeutic outcome through collaborative photothermal/radiation therapy under the guidance of NIR-II photoacoustic imaging. Moreover, CN-RB NPs demonstrate minimal side effects and long-term biological stability after 14 days. Therefore, the proposed new multifunctional nano-platform CN-RB NPs hold great potential in the application of deep therapeutics.

## Introduction

Photoacoustic (PA) imaging-guided photothermal therapy (PTT), which combines diagnosis with treatment in a single system, has attracted worldwide attention in recent years (Li et al., 2015; Sun et al., 2017; Cao et al., 2019). Specifically, owing to the reasonable sensitivity, high resolution up to the micrometers scale and deep tissue penetration up to the centimeter scale, PA imaging has great potential for biomedical diagnosis in clinical application (Wang, 2009; Zhang et al., 2010a; Hui et al., 2016; Liu and Qin, 2017). PTT utilizes photosensitizers to produce hyperthermia without oxygen under near-infrared (NIR) light irradiation, leading to hypoxia tumor ablation, which has spatiotemporal controllability to realize precise treatment (Huang et al., 2006). Compared with the NIR-I window (750–1000 nm), light in NIR-II (1000–1700 nm) has deeper tissue penetration and larger maximum permissible exposure (Bashkatov et al., 2005; Tian et al., 2011; Chen et al., 2013; Yong et al., 2014), suggesting that NIR-II laser-induced PTT is a more promising strategy for tumor ablation. However, NIR-II therapeutic agents have been rarely reported (Wu et al., 2015; Yang et al., 2017; Cao et al., 2018). Meanwhile, the treatment temperature is another considerable factor in the process of PTT. In order to obtain excellent therapeutic effects, the temperature of the photothermal agent must exceed 50°C so as to overcome the heat resistance of heat shock protein (HSP)^2^. Such a high temperature is bound to cause damage to normal organs and tissues and bring unbearable pain to patients in clinical treatment. Therefore, continued efforts have been devoted to combining NIR-II PTT with other therapeutic methods, expecting to ensure the depth and efficiency of treatment with appropriate temperatures. For example, in 2017, Chen et al. (2017) used plasmonic gold nanorods (GNRs), hyaluronic acid (HA) and Glut1 inhibitor of diclofenac (DC) to synthesize a GNR/HA-DC nano-platform, which improved the effect of mild PTT by inhibiting HSP overexpression. In 2020, Zhang et al. (2020) increased the therapeutic effect of NIR-II PTT by combining red-light-irradiated photodynamic therapy (PDT) through a TAT-Pd@Au/Ce6/PAH/H-MnO_2_ nano-platform. Synergistic therapy can improve the therapeutic effect. However, dual laser excitation creates additional complexity among the whole system.

Radiotherapy (RT) is an alternative tumor treatment in the clinic that can penetrate tissues deeply and kill deep tumor cells by inducing oxidative stress and/or destroying nuclear DNA (Horsman and Overgaard, 2007; Davis et al., 2015; Zhou et al., 2015; Huo et al., 2017; Shen et al., 2017; Tang et al., 2019). However, high doses of X-rays will inevitably cause damage to normal tissues (Bentzen et al., 2010), and the therapeutic effect of RT is limited to the tumor hypoxic microenvironment (Sun et al., 2008). Interestingly, previous reports found that the photothermal effect can improve the blood flow rate (Tang et al., 2019), hence increasing the oxygen level in the tumor cells which will promote the RT’s therapeutic effect. Therefore, considerable attention has been directed towards using PTT in combination with RT to reduce side effects caused by high dose of radiation and HSP of PTT (Ma et al., 2017). Currently, nano-platforms that have been applied in RT/PTT collaborative treatment are mainly inorganic semiconductors such as WS_2_ (Wang et al., 2019), MoS_2_/Bi_2_S_3_ (Wang et al., 2015), MnSe@Bi_2_Se_3_ (Song et al., 2015), and W-TiO_2_ (Gao et al., 2019). In 2019, Gao et al. synthesized a tungsten-doped titanium dioxide (W-TiO_2_) system to realize imaging and cooperative NIR-II PTT/RT therapy, which greatly improved the therapeutic effect (Gao et al., 2019).

Graphite-phase carbon nitride (g-C_3_N_4_), as a versatile inorganic semiconductor material, has been widely used in phototherapy (Chen et al., 2015; Ju et al., 2016). Our recent study found that g-C_3_N_4_ has a weak radiation therapeutic effect under X-ray irradiation. However, the absorption of g-C_3_N_4_ is limited to the visible region, which severely restricts the application of g-C_3_N_4_ in NIR phototherapy. It has been reported that the absorption of g-C_3_N_4_ increases in the visible and near-infrared regions after copolymerizing with carbon-rich materials or iodine doping (Zhang et al., 2010b; Zhang et al., 2014; Zheng et al., 2016). Moreover, the iodine element has also been proven to be a radiosensitizer, which can improve the efficacy of RT and reduce potential side effects. In 2015, Yi et al. synthesized iodine-doped copper sulfide nanoparticles to simultaneously realize imaging-guided synergetic PTT/RT (Ku et al., 2012). In 2020, Iqbal et al. reported the as-synthesized iodine doped mesoporous g-C_3_N_4_ demonstrated an outstanding photocatalytic H_2_ evolution performance of 7819.2 μmol h^−1^g^−1^ under simulated solar light irradiation, nearly 6.5 folds higher than that of the bulk g-C_3_N_4_ and other typical doped g-C_3_N_4_ photocatalysts (Iqbal et al., 2020).

In this study, inspired by the above, a new type of g-C_3_N_4_ (CN-RB NPs) was synthesized *via* the copolymerization of melamine and iodine-containing organic molecules. The enhanced absorption of CN-RB NPs in the NIR-II region presents an outstanding photothermal therapy effect with considerable photothermal conversion coefficient (*η* = 35%). Accordingly, CN-RB NPs showed strong PA signal intensity in the NIR-II window due to the excellent NIR-II photothermal effect. In addition, both *in vitro* and *in vivo* results demonstrated that CN-RB NPs preserved the efficient radiosensitive effect. Therefore, CN-RB NPs can provide NIR-II PTT/RT co-therapy with the guidance of NIR-II PA imaging, which can realize the integration of diagnosis and treatment simultaneously. Furthermore, CN-RB NPs showed low biological toxicity and long-term biological stability *in vivo*. Our research will provide a new avenue for the development of multifunctional theranostic agents for NIR-II PA imaging-guided PTT/RT co-therapy.

## Results and discussion

### Characterization of CN-RB NPs

**FIGURE 1:**
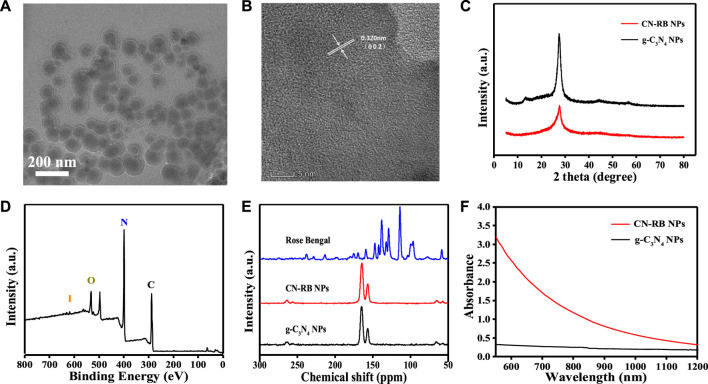
**(A)** TEM image of CN-RB NPs. **(B)** HRTEM of CN-RB NPs. **(C)** XRD patterns of CN-RB NPs and g-C_3_N_4_ NPs. **(D)** XPS survey spectrum of CN-RB NPs. **(E)** Solid-state NMR 13C {1H} spectra of CN-RB NPs and g-C_3_N_4_ NPs. **(F)** UV-vis absorption spectra of CN-RB NPs and g-C_3_N_4_ NPs.

In this experiment, modified CN-RB was synthesized by the thermal copolymerization of melamine and rose bengal (Zhang et al., 2010b). Then, a cell disruptor was used to convert make CN-RB into smaller CN-RB nanoparticles (CN-RB NPs). As indicated by the X-ray diffraction (XRD) spectra (Figure 1C), CN-RB NPs have the same <002> diffraction peak as g-C_3_N_4_ NPs at 27.6°, which can be ascribed to the stacking of aromatic structures (Zhang et al., 2017). In contrast, g-C_3_N_4_ NPs shows the <001> diffraction peak at 13.8°, while this does not exist in CN-RB NPs. The <001> diffraction peak is connected with in-plane structure stacking (Liu et al., 2011). The disappearance of the peak at 13.8° in CN-RB NPs is probably due to the change in the stacking structure in the process of copolymerization (Ho et al., 2015). As shown in the X-ray spectroscopy (XPS) spectra (Figure 1D), CN-RB NPs contain four elements of C, N, O and iodine I). The high-resolution image of C 1s (Fig. S1a, ESI†) shows three peaks at 288.3 eV, 285.7 eV, and 284.8 eV. The main peak of C 1s at 288.3 eV is sp^2^ hybrid carbon and the weaker peak at 284.8 eV corresponds to graphite-phase carbon (Liu et al., 2015). The high-resolution images of N 1s and O 1s are shown in Fig. S1b and S1c (ESI†). The peaks of N 1s are 398.8 eV and 399.6 eV, and the peaks of O 1s are 535.6 eV, 532.8 eV, and531.5 eV, respectively. In addition, the high-resolution peaks of I 3 days (Fig. S1d, ESI†) are seen at 630.1 eV and 619 eV (Zhang et al., 2014). The presence of I element can be confirmed from the XPS spectrum, but it does not appear in the XRD, probably due to the low content of I element. Supported by the Fourier transform infrared (FTIR) spectra in Fig. S2 (ESI†), the multiple bands of CN-RB NPs are located at 809 cm^−1^, 1200–1650 cm^−1^, and 3400 cm^−1^, which confirm that the copolymerized CN-RB NPs have the skeleton of the g-C_3_N_4_ NPs (Ho et al., 2015). In addition, the CN-RB NPs show two resolved signals in the solid-state NMR 13C {1H} cross-polarization spectrum (Figure 1E) at 157 ppm and 165 ppm, which are the same as in g-C_3_N_4_ NPs. The above results further indicate that the copolymerization of CN-RB NPs leads to an identical chemical skeleton to g-C_3_N_4_ NPs.

The hydrodynamic diameter of CN-RB NPs in aqueous solution (Fig.S3, ESI†) is approximately 130 nm, which is suitable for biological application (Wu et al., 2016). It can be seen from the transmission electron microscopy (TEM) image (Figure 1A) that the particle size of CN-RB NPs is approximately 100 nm, which is slightly smaller than the size of the hydrodynamic diameter. As shown in Figure 1B, the high-resolution transmission electron microscopy (HRTEM) image suggests that the lattice spacing of CN-RB NPs is 0.340 nm, which indicates that CN-RB NPs have a graphene-like structure (Stankovich et al., 2007). Figure 1F shows that the absorption of CN-RB NPs in the NIR-II window is significantly enhanced, which indicates that CN-RB NPs can be used as a NIR-II window photothermal agent. In order to verify that the enhanced absorption is not solely induced by the carbon-rich molecular structure, the absorption of CN-B NPs is compared with CN-LB NPs synthesized by melamine and rhodamine B, as they have a similar structure to rose bengal. The results (Fig. S4, ESI†) show that the absorption of CN-RB NPs in the NIR region is much stronger than that of CN-LB NPs, confirming that the doping of I element indeed contributes to the increased absorption of CN-RB NPs in the NIR region. The above results all suggest that the obtained CN-RB NPs not only maintain the skeleton of g-C_3_N_4_, but also enhance the optical absorption in the NIR-II window, which will lay a solid foundation for their application in biomedicine.

### NIR-II photothermal effect of CN-RB NP solution

**FIGURE 2:**
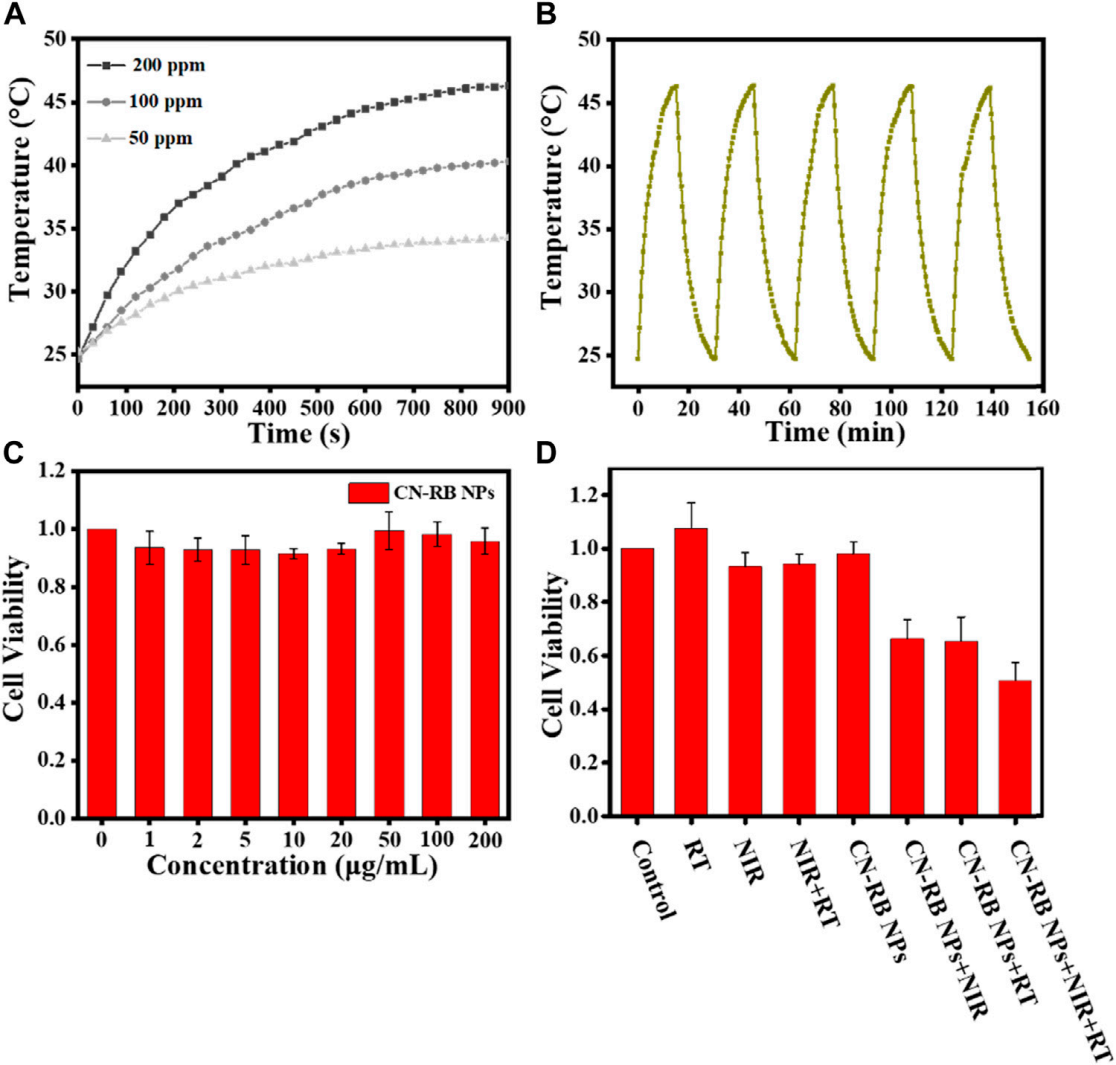
**(A)** The photothermal effect of different concentrations of CN-RB NPs solution under 1064 nm laser irradiation (2 W cm^−2^). **(B)** The photothermal effect of CN-RB NPs solution of 200 ppm over five cycles under 1064 nm laser irradiation (2 W cm^−2^). **(C)** Cell viability of 4T1 breast cancer cells exposed to CN-RB NPs at different concentrations (n = 5, *p* < 0.05). **(D)** Relative cell viability of each group evaluated using MTT assay (*n* = 5, *p* < 0.05).

The absorbance of CN-RB NPs is extended from visible light to the NIR window (>1000 nm), suggesting that CN-RB NPs have potential for NIR-II PTT. As the absorption of CN-RB NPs has no sharp peaks, we choose the 1064 nm laser to trigger NIR-II PTT. We first tested the photothermal properties of CN-RB NPs at different concentrations (50 ppm, 100 ppm and 200 ppm). Figure 2A shows that under 1064 nm laser irradiation for 15 min, the increased temperature of the CN-RB NPs solution is dependent on the increasing concentration. At the concentrations of 50 ppm and 100 ppm, the temperature of the CN-RB NP solution can increase to 34.3°C and 40.3°C, respectively. When the concentration is 200 ppm, the temperature of CN-B NPs can rise to 46.3°C, which is suitable for mild-temperature photothermal therapy (Liu et al., 2017). Next, the photothermal stability of CN-RB NPs was measured in a cycle experiment. CN-RB NPs solution (200 ppm) was first irradiated with a 1064 nm laser (2 W cm^−2^) for 15 min, and then the solution was allowed to cool to the original temperature (24.7°C). As shown in Figure 2B, after five cycles, the maximum temperature of the CN-RB NP solution did not change in each cycle, indicating that CN-RB NPs have good thermal stability. The photothermal conversion efficiency η) of CN-RB NPs was calculated to be 35%, which is comparable to some nanomaterials with NIR-II mild photothermal effects (Yu et al., 2020). The above results suggest that CN-RB NPs can be used as an NIR-II photothermal agent.

### *In vitro* cytotoxicity of CN-RB NPs

Here, the cell cytotoxicity of CN-RB NPs was appraised with an MTT assay. The cell viability of 4T1 cells incubated with different concentrations (0, 1, 2, 5, 10, 20, 50, 100, and 200 μg mL^−1^) of CN-RB NPs for 24 h was above 95% in Figure 2C. It indicates that there is no obvious cellular death produced by CN-RB NPs in the dark.

### *In vitro* NIR-II photothermal therapy and radiation therapy of CN-RB NPs

To estimate the *in vitro* therapeutic efficiency of CN-RB NPs, the MTT assay was performed to determine the cell viability under different experimental conditions. It can be seen from Figure 2D that the survival rate of 4T1 cells treated with X-ray alone and a 1064 nm laser alone was 96% and 98%, respectively. In addition, we considered the radiation therapy of g-C_3_N_4_ NPs. As shown in Fig. S5, the death rate of cells co-incubated with g-C_3_N_4_ NPs is approximately 10% after X-ray irradiation, indicating that g-C_3_N_4_ NPs have a weak response to X-ray. However, when treated with the PTT or RT of CN-RB NPs, the survival rate of 4T1 cells can be reduced to 65% and 62%, respectively, which suggests that CN-RB NPs not only have NIR-II PTT capability, but can be used for RT. With PTT and RT co-therapy, the cell viability decreases to 45%, confirming the *in-vitro-*enhanced PTT/RT co-therapy. The *in vitro* cells experiment encourages us to carry out further animal experiments.

### *In vivo* infrared thermal/photoacoustic imaging

**FIGURE 3:**
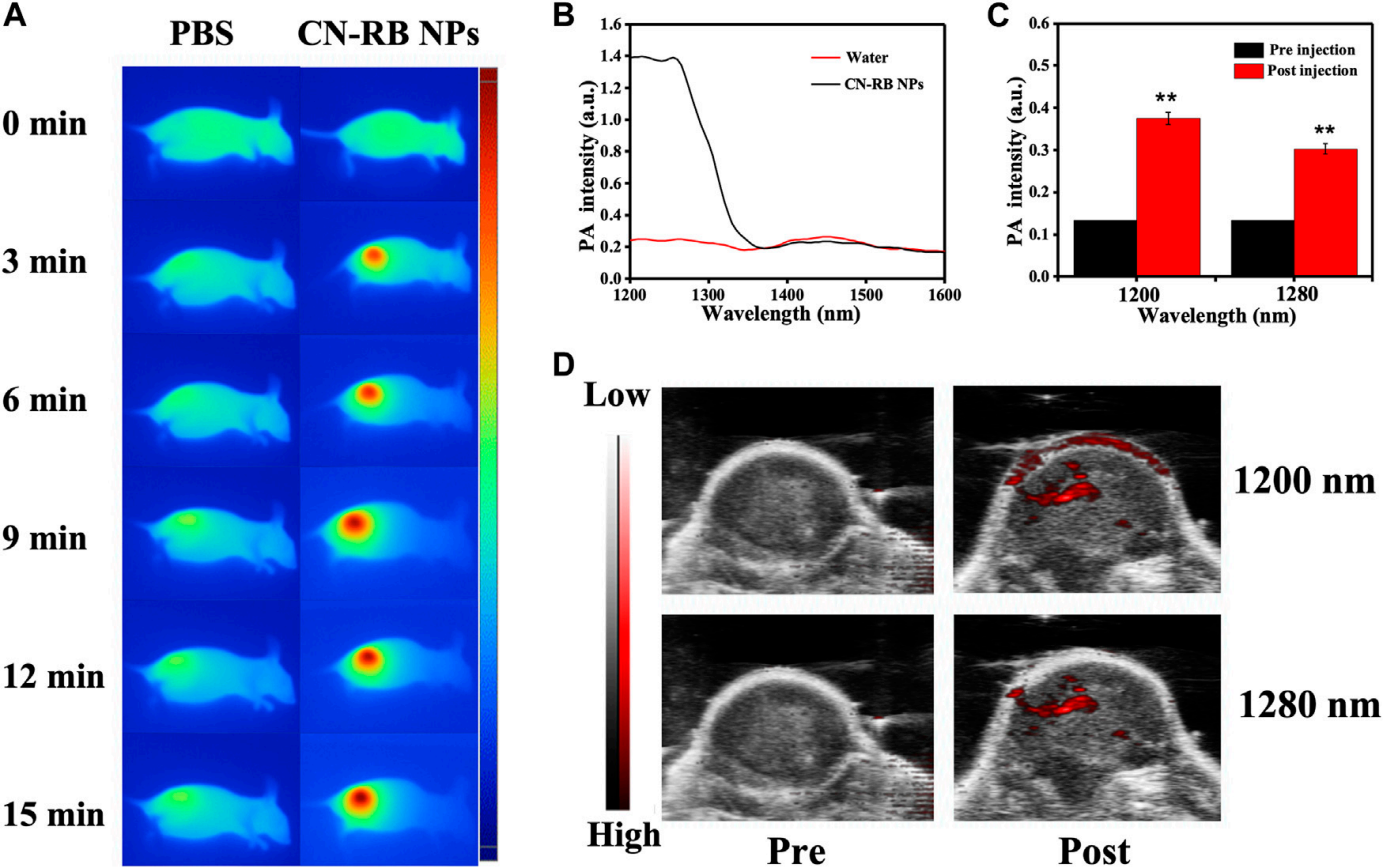
**(A)** Infrared thermal imaging of 4T1-tumor-bearing BLAB/c nude mice injected with PBS or CN-RB NPs after irradiation at 1064 nm (2 W cm^−2^) for 15 min **(B)** *In vitro* PA signal of CN-RB NP solution from 1200 to 2000 nm. **(C)** At 1200 nm and 1280 nm, *in-vivo-*quantified PA signal of tumors pre-injection and post-injection of CN-RB NPs. **(D)** *In vivo* PA images pre-injection and post-injection of CN-RB NPs at 1200 nm and 1280 nm.

Based on the *in vitro* NIR-II PTT/RT co-therapy of CN-RB NPs, we studied the tumor-suppressive effect of the CN-RB NPs in 4T1-tumor-bearing BALB/c nude mice. In order to evaluate the photothermal effect of CN-RB NPs in the NIR-II window, we used an infrared thermal imager to record the temperature of the tumor site in mice injected with CN-RB NPs under 1064 nm laser irradiation (2 W cm^−2^). It can be seen from Figure 3A that after being irradiated for 15 min, the temperature of the tumor site of the mouse could reach 45 °C, while the temperature of the tumor site of the mice injected with PBS hardly changed. The above data confirm that CN-RB NPs have a good photothermal effect under 1064 nm laser irradiation. PA imaging is widely used in biomedicine due to its high resolution and deep penetration. We first measured the PA signal of the CN-RB NPs solution and pure water from 1200 to 2000 nm. Figure 3B shows that in the range of 1200–1280 nm, the quantified PA signal of CN-RB NPs is 7 times stronger than that of pure water. At the wavelength of 1300 nm, the quantified PA signal of CN-RB NPs is 4 times stronger than that of pure water. CN-RB NPs have a strong PA signal in the NIR-II window. Furthermore, we evaluated the PA signal of CN-RB NPs *in vivo* in tumor tissues. Figures 3C,D show that, in contrast to the case before injection, the PA signal at 1200 nm and 1280 nm in the tumor tissues injected with CN-RB NPs was increased by 2.7 times and 2 times, respectively, indicating that CN-RB NPs are suitable for NIR-II PA imaging.

### NIR-II photothermal/radiation co-therapy of CN-RB NPs

**FIGURE 4:**
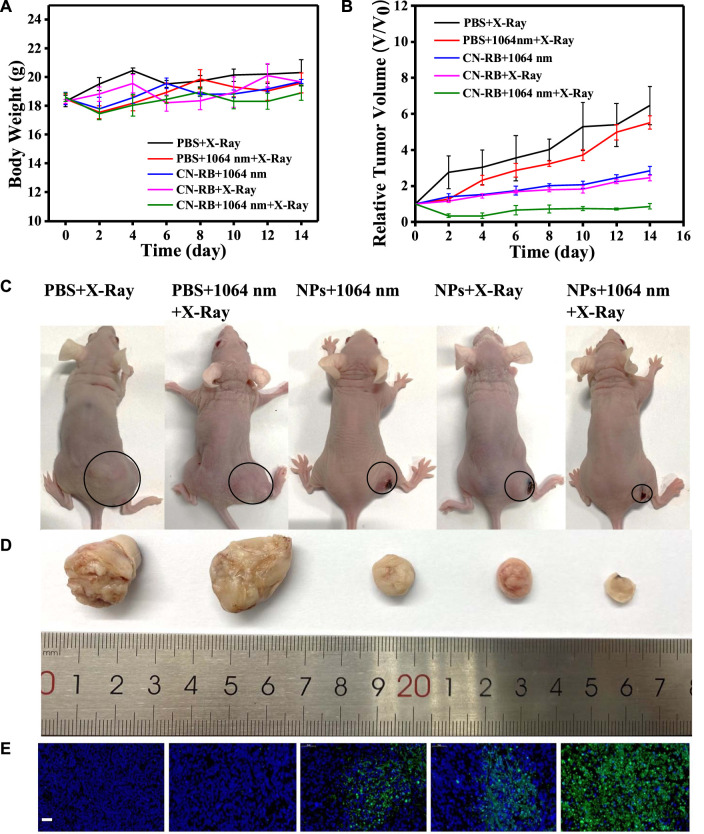
**(A)** Body weights of 4T1-tumor-bearing BALB/c nude mice with different treatments. **(B)** Tumor volumes of 4T1-tumor-bearing BALB/c nude mice with different treatments. **(C)** Digital photographs of five groups of 4T1-tumor-bearing BLAB/c nude mice and tumors are circled (*n* = 3). **(D)** Corresponding images of excised tumors from 4T1-tumor-bearing BLAB/c nude mice after 14 days treatment. **(E)** TUNEL assay of tumor slices of different groups after 14 days, green fluorescence represents apoptotic cells. (the scale bar is 50 μm).

**FIGURE 5:**
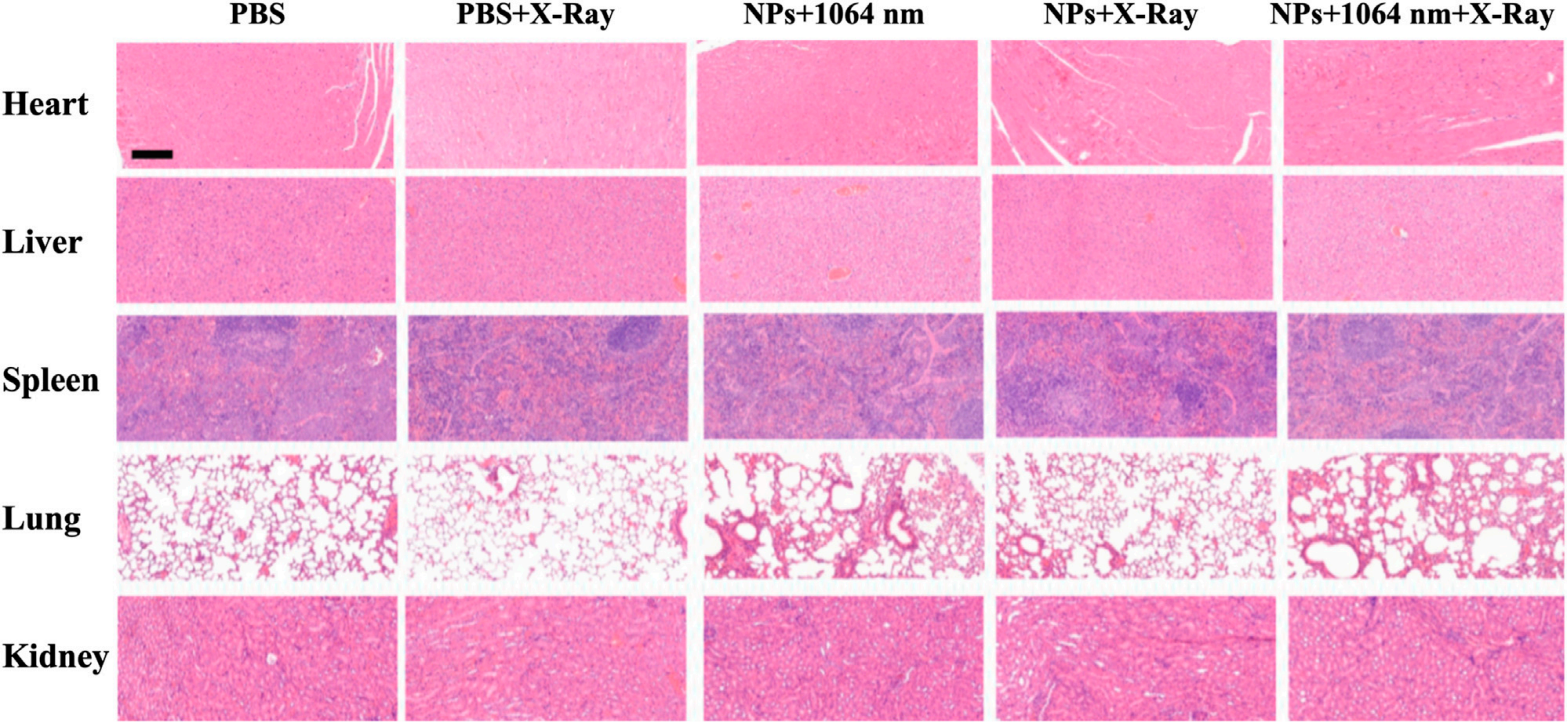
H&E staining of different organs of 4T1-tumor-bearing BLAB/c nude mice after 14 days. The scale bar is 50 μm.

To evaluate the therapeutic efficiency *in vivo*, the mice were divided into five groups (PBS, PBS + RT, CN-RB NPs+1064 nm, CN-RB NPs + RT, and CN-RB NPs+ 1064 nm + RT, respectively, *n* = 3). The first group (PBS+1064 nm) and the second group (PBS+1064 nm + RT) were the control groups. Mice in the control groups were intratumorally injected with PBS, and then irradiated with 1064 nm and/or X-ray. Mice of the third group (CN-RB NPs+1064 nm) and the fourth group (CN-RB NPs + RT) were intratumorally injected with CN-RB NPs and then irradiated with a 1064 nm laser or X-ray. Mice in the fifth group (CN-RB NPs+1064 nm + RT) were intratumorally injected with CN-RB NPs, first irradiated with a 1064 nm laser, and then irradiated with X-ray. As shown in Figure 4A, there were no obvious loss of body weight or death in all groups, which indicate that CN-RB NPs have no significant toxicity. It can be seen from Figure 4B that, after 14 days, the tumors of the mice in the first and second groups grew rapidly. In contrast, the tumors in the third, fourth, and fifth groups were suppressed. Among them, the tumor inhibition effect in the third group of mice and the fourth group of mice was equivalent, which showed that CN-RB NPs not only had a photothermal treatment effect under 1064 nm laser irradiation, but also had a radiotherapy effect under X-ray irradiation. At the same time, the tumor suppression effect in the fifth group of mice was the most obvious compared with the other four groups, which indicated the enhancement caused by the NIR-II PTT/RT co-therapy of CN-RB NPs. After 14 days, the tumor tissues were removed from the mice. As can be seen from Figures 4C,D, the tumor tissues of mice in the treatment group were much smaller than those in the control groups. The temperature of tumor site of the mouse after 15 min’ photothermal/radiotherapy can reach to 45°C, this may cause a little bit skin burnt after treatment. However, the main purpose is removing malignant tumor (in our experiment, breast cancer) completely. Aesthetic problem is not our primary concern. We think a little superficial burning scar is acceptable. Moreover, among the treatment groups, the therapeutic efficiency of the NIR Ⅱ PTT/RT co-therapy group was the best. We also estimated the therapeutic therapy of CN-RB NPs by TUNEL assay. As shown in Figure 4E, the group that underwent NIR-II PTT/RT co-therapy showed the most apoptotic cells, which indicates that synergistic treatment can achieve a better therapeutic effect. To further consider the side effects of the long-term accumulation of CN-RB NPs, we next estimated the potential toxicity of CN-RB NPs *in vivo*. After 14 days, the hearts, livers, spleens, lungs, and kidneys of all five groups of mice were collected and stained with hematoxylin and eosin (H&E). There was no obvious pathological damage in all organs (Figure 5), which suggests that CN-RB NPs exhibit low toxicity *in vivo* and can be used as a long-term therapeutic agent. The all above results demonstrate that CN-RB NPs can be applied in NIR-II PTT/PT co-therapy and have long-term biostability.

## Materials and methods

### Material

Melamine and rose bengal were purchased from Aladdin. DCFH-DA and 3-(4,5-dimethyl-2-thiazolyl)-2,5-diphenyl-2-H-tetrazolium bromide (MTT) were purchased from Sigma-Aldrich. RPMI 1640 medium and Sciecell fetal bovine serum were purchased from Hyclone. Trypsin—EDTA (0.25%), dimethyl sulfoxide (DMSO), penicillin and streptomycin were purchased from Gibco. All chemical reagents were of analytical grade and were used as received without further purification.

### Synthesis of CN-RB NPs

CN-RB NPs were synthesized through one-step thermal copolymerization (Zhang et al., 2010b). Briefly, 1.0 g of melamine and rose bengal in a ratio of 1:1 was ground in a mortar for 30 min and then calcined in a muffle furnace at 520°C for 4 h, with a heating rate of 5°C per min. After cooling to room temperature, the obtained sample was in the form of CN-RB. Next, 500 mg of CN-RB was added to 50 mL of ultra-pure water and crushed in an ultrasonic cell breaker in an ice bath for 30 min (power was 500 W, working for 3 s, 6 s apart). The suspension was centrifuged at 8000 rpm for 10 min to remove unexfoliated CN-RB. Finally, the collected supernatant was labelled as CN-RB NPs and was stored in the refrigerator at 4°C for future use.

### Instruments

The morphology and size of the CN-RB NPs were measured through transmission electron microscopy (Talos F200X G2). XRD was performed using an XRD-7000 (Shimazdu, Japan). X-ray photoelectron spectroscopy (XPS) spectra were obtained using an AXIS UltraDLD (Shimazdu, Japan). The UV—vis absorption was measured using a UV-1800 spectrophotometer (Shimadzu, Japan). Photoacoustic imaging was performed using a photoacoustic imager (Fujifilm VisualSonics/VEVO LAZR-X) and X-ray irradiator (Rad Source).

### Photothermal conversion efficiency η) of CN-RB NPs

The photothermal conversion efficiency ( η ) was measured by the following Equation 1: (Li et al., 2019)

$$\eta =\frac{hA\left(T_{2}-T_{1}\right)-Q_{1}}{I\left({1-10}^{{-A}_{\lambda }}\right)}$$ (1)

h : heat-transfer coefficient;

A : surface area of the container;

$T_{2}$ : the equilibrium temperature;

$T_{1}$ : the ambient temperature;

$Q_{1}$ : the heat dissipation from the quartz cell;

I : laser power;

$A_{\lambda }$ : the absorbance of CN-B NPs at wavelength of 808 nm.

The hA value was calculated by the following Equation 2 (Ku et al., 2012):

$$hA=\frac{mC_{P}}{\tau_{s}}$$ (2)

m : the solution mass;

$C_{P}$ : the heat capacity of water;

$\tau_{s}$ : the sample time constant.

### *In vitro* cell cytotoxicity of CN-RB NPs

To evaluate the cytotoxicity of the CN-RB NPs in 4T1 cells, 4T1 mouse breast cancer cells were seeded in a 96-well plate (5×10^3^ cells mL^−1^) and were incubated with different concentrations of CN-B NPs (0, 1, 2, 5, 10, 20, 50, 100, and 200 μg mL^−1^) for 24 h at 37°C and 5% CO_2_. After 24 h, 10 µL of MTT solution (5 mg mL^−1^ diluted in PBS) was added into each well. After 4 h incubation at 37 °C, the culture medium was discarded and 100 µl of DMSO was added to dissolve the formed formazan crystals. The amount of living cells was measured using an enzyme mark instrument at 490 nm.

### Evaluation of *in vitro* NIR-II PTT/RT co-therapy with MTT assay

First, 4T1 mouse breast cancer cells (5×10^3^ cells mL^−1^) were seeded in 96-well plates. The medium was replaced after incubation at 37°C in a humidified environment with 5% CO_2_, 21% O_2_ for 24 h. The experimental sample was divided into eight groups (*n* = 6). The first four groups were control groups. The first group was the cell control group; the cells in the second group and the third group were irradiated by a 1064 nm laser and X-ray, respectively. The cells in the fourth group were first irradiated by a 1064 nm laser and then X-ray. The last four groups were the experimental groups. The fifth group was only incubated with CN-RB NPs (200 μg mL^−1^) for 4 h and the sixth group was first incubated with CN-RB NPs (200 μg mL^−1^) for 4 h and then irradiated with a 1064 nm laser for 15 min. The seventh group was first incubated with CN-RB NPs (200 μg mL^−1^) for 4 h and then irradiated with X-ray. After incubation with CN-RB NPs (200 μg mL^−1^) for 4h, the eighth group was irradiated by a 1064 nm laser for 15min and then irradiated by X-ray. After continuing to cultivate for 20 h, 10 µl of MTT (5 mg mL^−1^) was added to each well for all groups. The medium was removed after 4 h incubation at 37°C and 100 µl of DMSO was added to dissolve the formazan crystals. The absorption at 490 nm was measured using an enzyme mark instrument.

### *In vivo* infrared thermal imaging of CN-RB NPs

The 4T1 mouse breast cancer cells were inoculated in the right underarm of the 4T1-bearing-tumor BALB/c mice. When the tumor volume reached 100 mm^3^, 100 µl CN-RB NPs (300  μg mL^−1^) solution was injected into the tumor tissues of the mice. Then, infrared thermal imaging was performed using an infrared thermal imager at different time points with 1064 nm laser irradiation.

### *In vivo* PA imaging of CN-RB NPs

The 4T1 mouse breast cancer cells were inoculated in the right underarm of the mice. When the tumor volume reached 100 mm^3^, 100 µL of a CN-RB NP solution (300 μg mL^−1^) was injected into the tumor tissues of the mice. PA imaging of the tumor site was performed using a photoacoustic imager.

### *In vivo* NIR-II PTT/RT co-therapy using CN-RB NPs

Based on the results of *in vitro* experiments, the therapeutic efficiency of CN-RB NPs was evaluated in 4T1-tumor-bearing BALB/c nude mice. All animal operations were performed under protocols approved by the Animal Experiment Center of Shanghai Jiao Tong University. When the average tumor volume of the mice reached approximately 100 mm^3^, the mice were randomly divided into four groups (*n* = 3). The first group and the second group were intratumorally injected with 100 µl of PBS, and the third, fourth and five groups were intratumorally injected with 100 µl of CN-RB NPs (300 μg mL^−1^). The second group and the fourth group were irradiated only with X-ray. The third group was irradiated only with a 1064 nm laser (2 W cm^−2^) for 15 min. The fifth group was irradiated with a 1064 nm laser for 15 min and then irradiated with X-ray. The concentration of CN-RB NPs was 2 mg kg^−1^. The body weight and tumor diameter (using a Vernier caliper) were measured every day, and the tumor volume was calculated as V = d^2^×D/2 (d was the shortest diameter of the tumor, and D was the longest diameter of the tumor).

### Statistical analysis

Graphpad Prism 7.0 statistical software (La Jolla, CA) was used for data analysis, and one-way analysis of variance (one-way ANOVA) was used for comparison among multiple groups. Data are expressed as mean ± standard deviation, *p* < .05 as well as *p* < .01 means the difference is statistically significant.

## Conclusion

In summary, iodine-containing and carbon-rich graphite carbon nitride (CN-RB) were synthesized by one-step thermal copolymerization. CN-RB NPs with a small particle size of less than 200 nm were obtained by ultrasonic breaking. The results show that the absorption of CN-RB NPs is strongly enhanced in the NIR-II window, and CN-RB NPs have a significant photothermal effect under 1064 nm laser irradiation, which indicates that CN-RB NPs can be used for NIR-II PTT. *In vitro* experiments show that g-C_3_N_4_ NPs have a certain radiation therapy capability under X-ray irradiation, and the radiation effect of CN-RB NPs doped with iodine is greatly enhanced. The NIR-II PTT/RT therapeutic effect is better than that of either NIR-II PTT or RT alone. In addition, CN-RB NPs also have an obvious PA signal in the NIR-II window (at 1200 nm and 1280 nm), which indicates that CN-RB NPs can be used for NIR-II tumor diagnosis. Subsequently, *in vivo* results show that CN-RB NPs not only can be applied in NIR-II PA imaging, but also have a significant inhibitory effect on tumors under the irradiation of a 1064 nm laser and/or X-ray. Based on the above experimental results, CN-RB NPs can simultaneously achieve NIR-II PA imaging and PTT/RT co-therapy.

## Data Availability

The original contributions presented in the study are included in the article/Supplementary Material, further inquiries can be directed to the corresponding authors.
