# Supplementary material for: Copolymerized carbon nitride nanoparticles for near-infrared II photoacoustic-guided synergistic photothermal/radiotherapy
Source: Front Chem. 2023 Jan 13;11:1124559. doi: 10.3389/fchem.2023.1124559 (PMC9880048; doi:10.3389/fchem.2023.1124559)
Supplement: Supplementary file 1 [file DataSheet1.docx]

Article

Copolymerized Carbon Nitride Nanoparticles for Near-Infrared II Photoacoustic-Guided Synergistic Photothermal/Radiotherapy

Min Wu^#^*^1^, Yuxin Huang^#3^, Xiaoyu Huang^3^, Fu Wang*^3^ and Xunbin Wei*^2^

^1^ Department of Plastic and Reconstructive Surgery, School of Medicine, Shanghai Ninth People’s Hospital, Shanghai Jiao Tong University, Shanghai, China; sjtu_wm@126.com

^2^ Biomedical Engineering Department, Peking University, Beijing, China; xwei@bjmu.edu.cn

^3^ School of Biomedical Engineering, Shanghai Jiao Tong University, Shanghai, China; wangfu@sjtu.edu.cn; xiaoyuhuang@sjtu.edu.cn; huangyuxin9999@163.com

***** Correspondence: xwei@bjmu.edu.cn; wangfu@sjtu.edu.cn


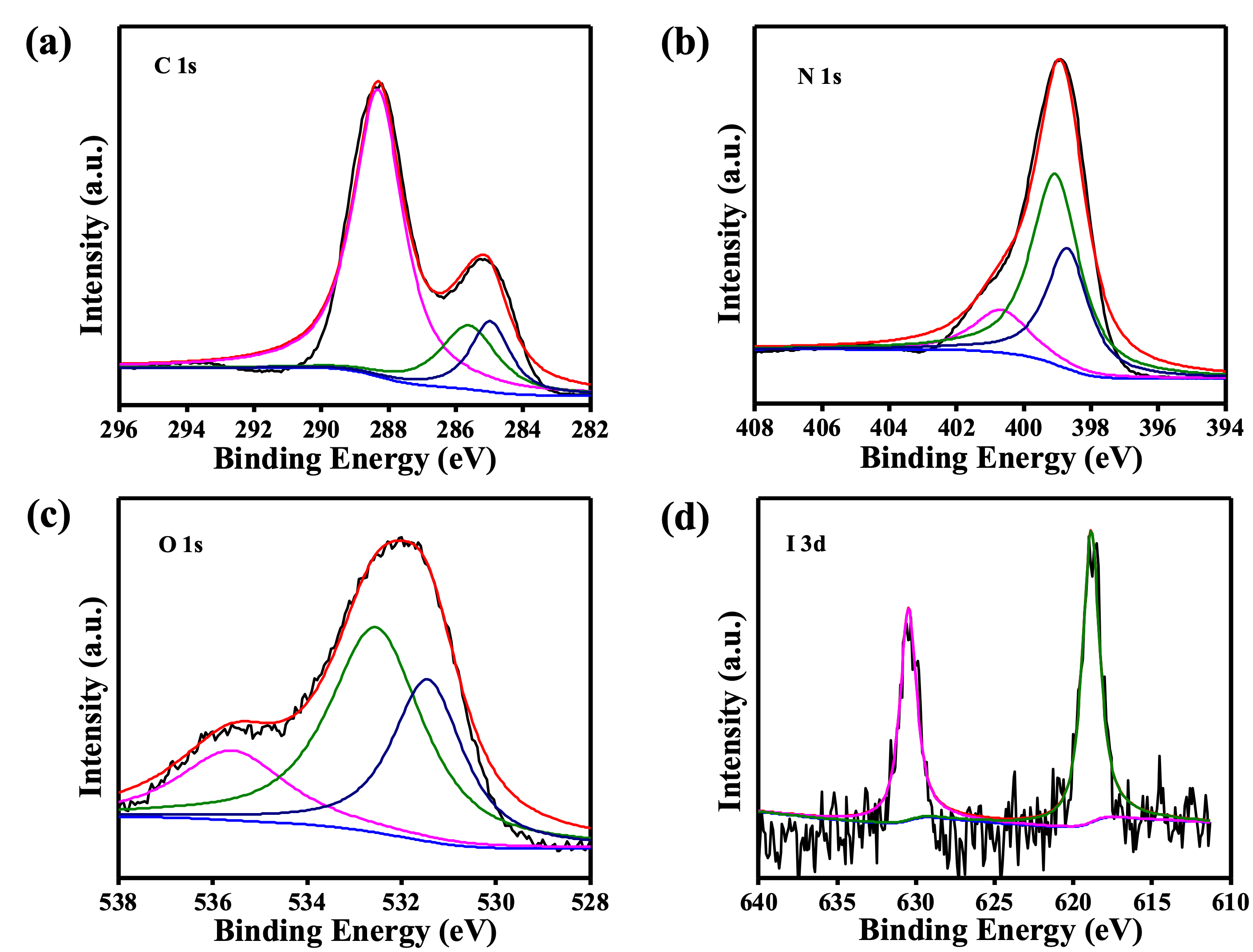


**Figure S1.** (a) High resolution of C 1s spectra of the CN-RB NPs. (b) High resolution of N 1s spectra of the CN-RB NPs. (c) High resolution of O 1s spectra of the CN-B NPs. (d) High resolution of I 3d spectra of the CN-RB NPs.


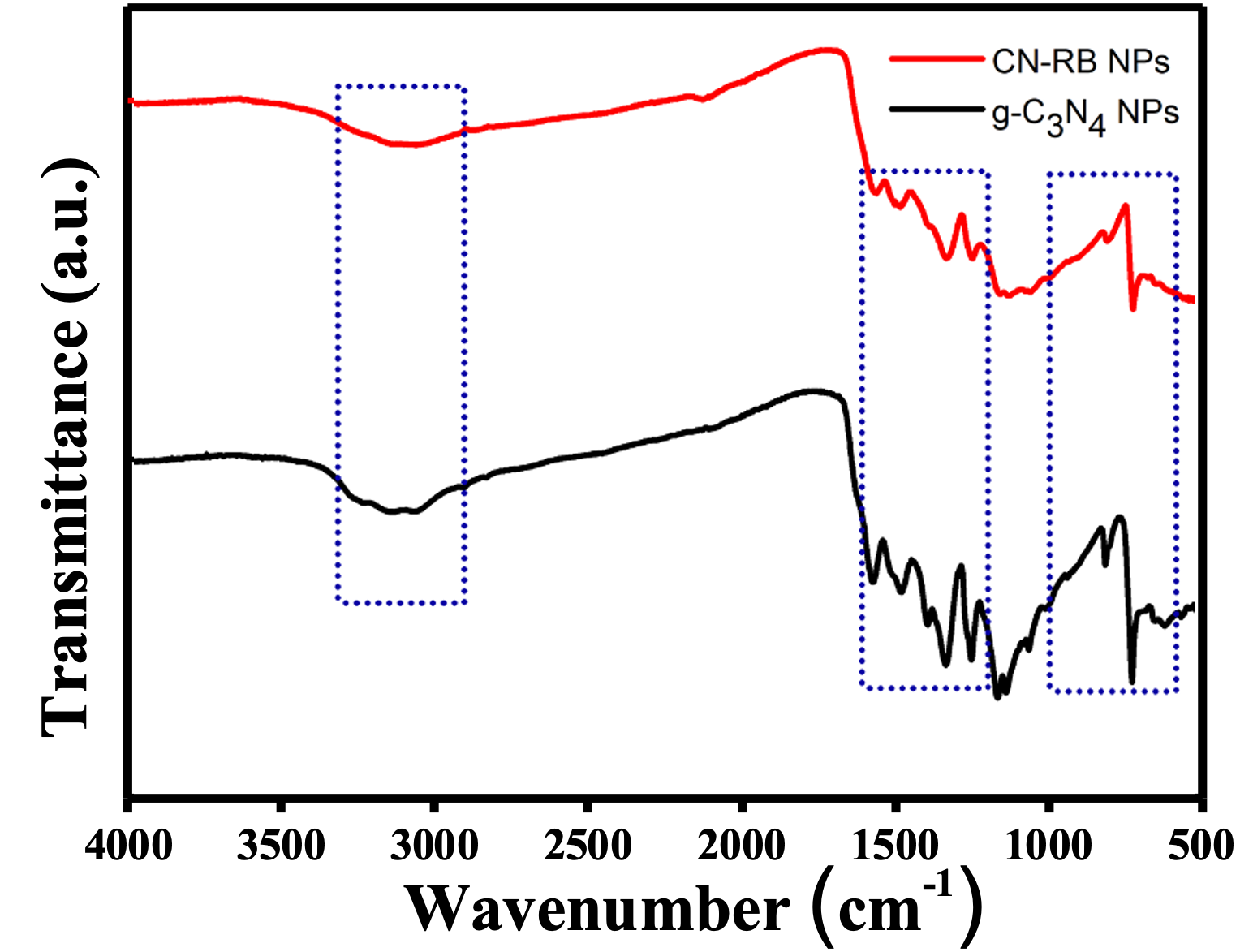


**Figure S2.** FTIR spectra of CN-RB NPs and g-C_3_N_4_ NPs.


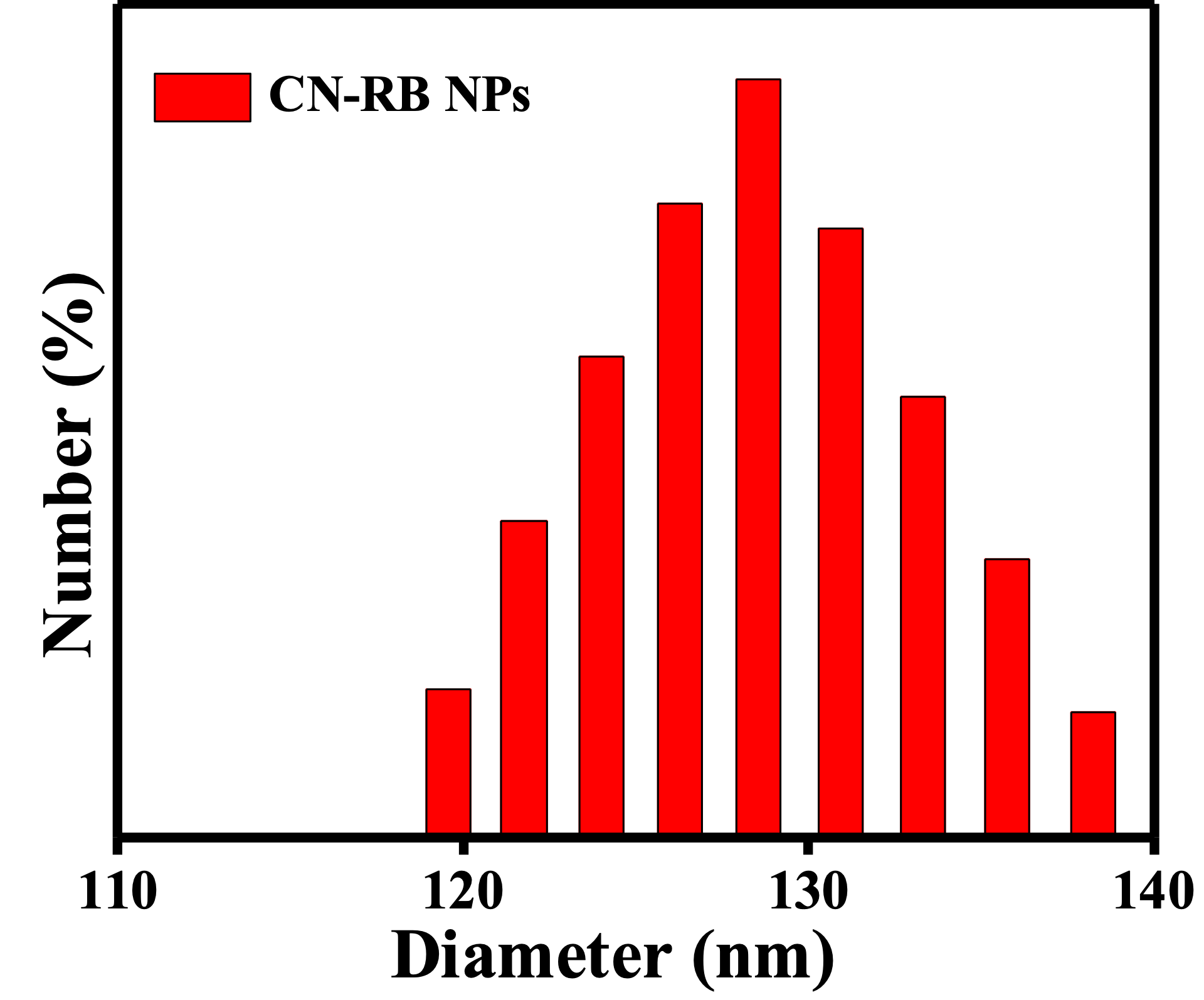


**Figure S3.** Size distribution of the CN-RB NPs.


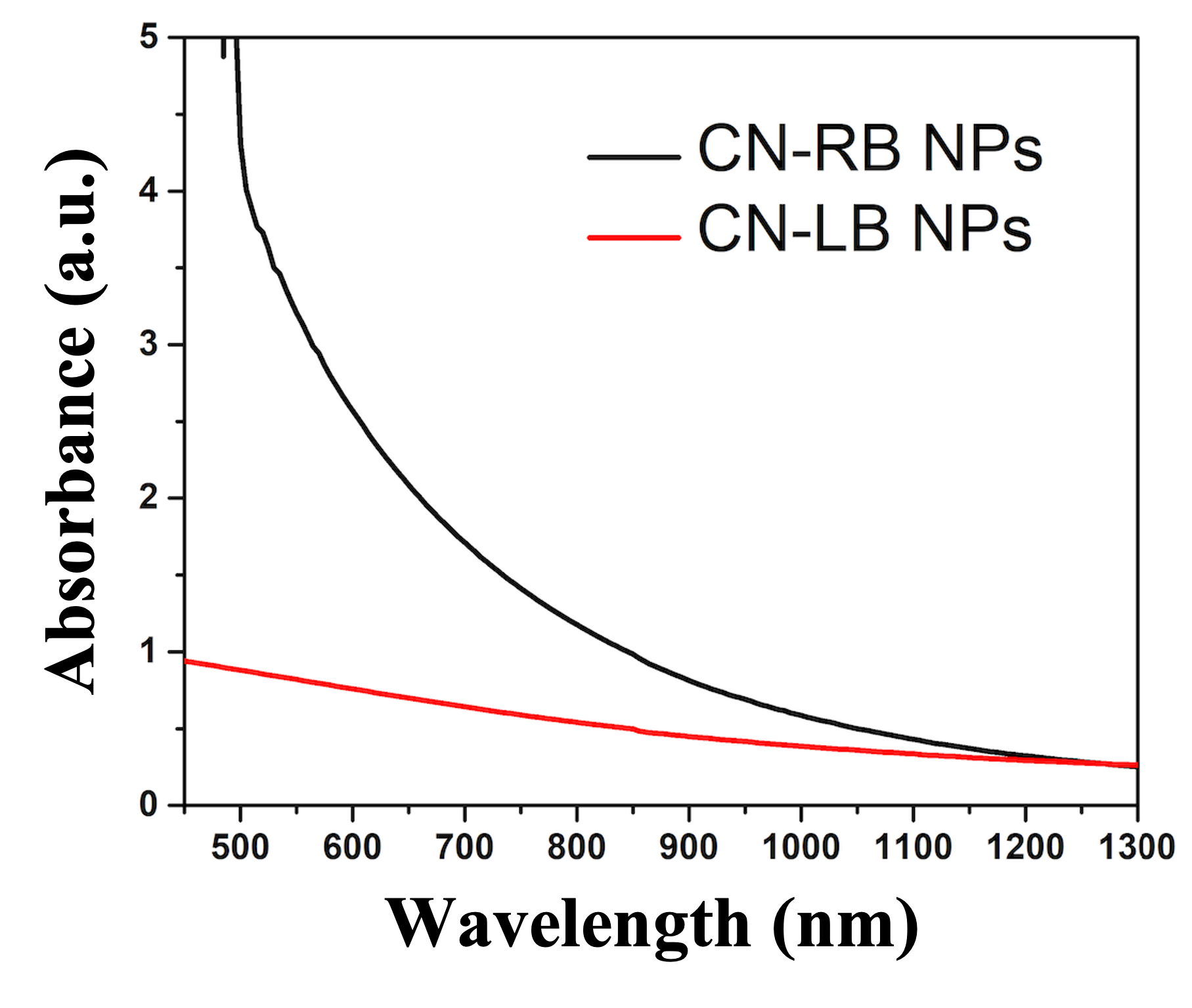


**Figure S4.** UV-vis absorption spectra of CN-RB NPs and CN-LB NPs.


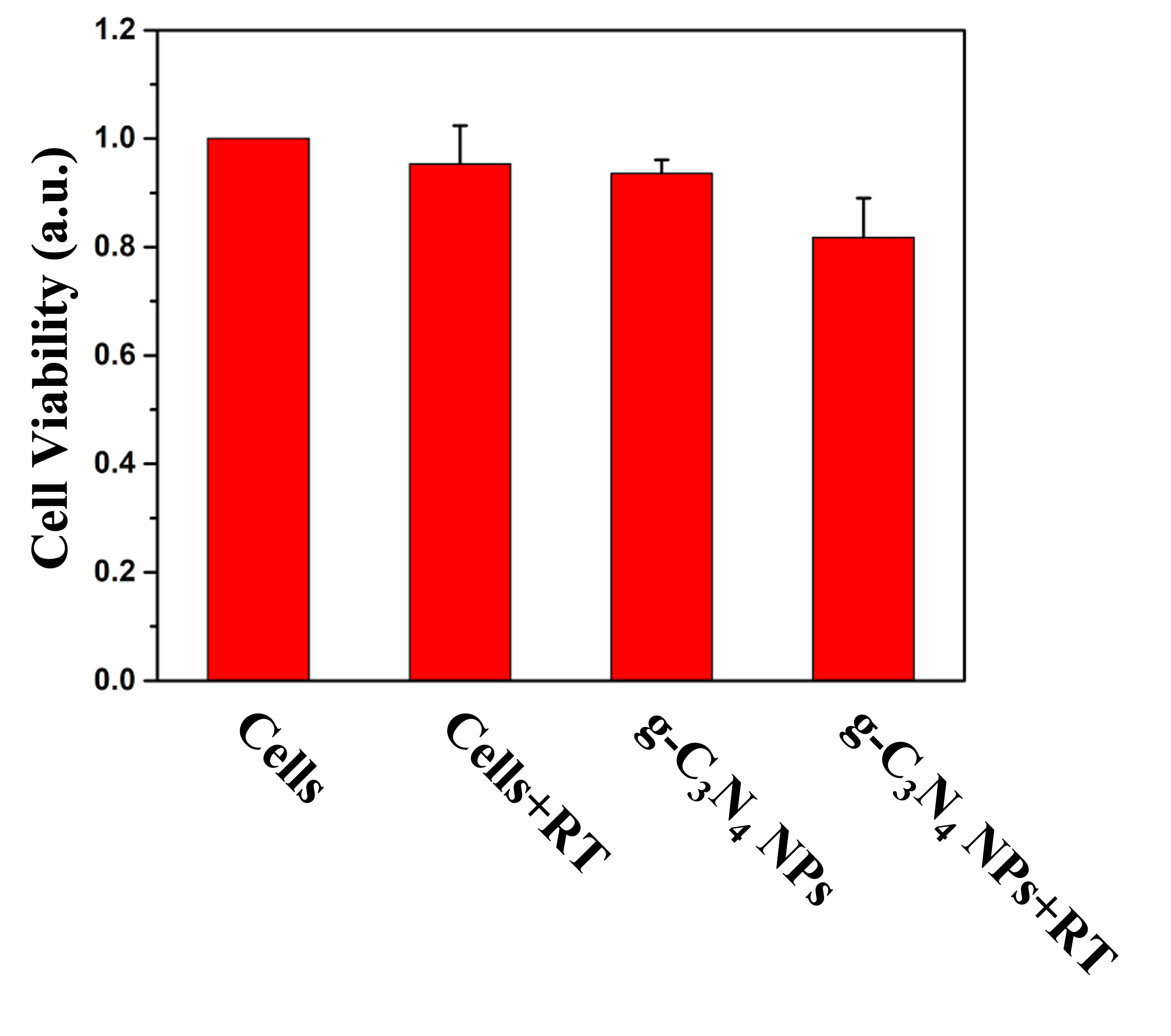


**Figure S5.** Relative cell viability of each group evaluated using MTT assay.
